# PPD-induced monocyte mitochondrial damage is associated with a protective effect to develop tuberculosis in BCG vaccinated individuals: A cohort study

**DOI:** 10.1371/journal.pone.0171930

**Published:** 2017-02-21

**Authors:** Diana Marín, Nancy Marín, Helena del Corral, Lucelly López, María Elena Ramirez-Agudelo, Carlos A. Rojas, María P. Arbeláez, Luis F. García, Mauricio Rojas

**Affiliations:** 1 Grupo de Epidemiología, Facultad Nacional de Salud Pública, Universidad de Antioquia, Medellín, Colombia; 2 Centro Colombiano de Investigación en Tuberculosis (CCITB), Colciencias, Medellín, Colombia; 3 Grupo de Inmunología Celular e Inmunogenética, Facultad de Medicina, Universidad de Antioquia, Medellín, Colombia; 4 Unidad de Citometría de Flujo, Sede de Investigación Universitaria, Universidad de Antioquia, Medellín, Colombia; Colorado State University, UNITED STATES

## Abstract

**Introduction:**

The mechanisms of mononuclear phagocyte death have been associated with the permissiveness and resistance to mycobacterial replication, but it remains unknown whether or not they help predict the risk of developing TB.

**Objective:**

To describe the factors associated with the induction of monocyte mitochondrial and membrane damage in response to PPD as well as determine if this type of damage might predict the susceptibility of developing active tuberculosis in a cohort of household contacts (HHCs) from Medellin, Colombia from 2005 to 2008.

**Methods:**

The prospective cohort study contains 2060 HHCs patients with pulmonary tuberculosis who were meticulously followed for two years. A survey of the socio-demographic, clinical, epidemiological factors and blood samples were collected. Mononuclear cell cultures were stimulated with or without PPD and the type of monocyte death was determined by the flow of cytometry, an indicator was also used for its analysis. Logistic regression was adjusted by the Generalized Estimations Equations and the survival was estimated with the Kaplan-Meier and Cox regression. Confidence intervals were used for estimating the association.

**Results:**

1,859 out of 2,060 blood samples of the HHCs patients analyzed showed monocyte death. In response to PPD, 83.4% underwent mitochondrial damage while 50.9% had membrane damage. The membrane damage in response to PPD was higher in children under 4 years (OR: 1.57; (95% CI: 1.1 to 2.4) and the HHCs who slept regularly in the same household has an index case of (OR: 1.54; 95% CI: 1.0 to 2.3). After adjustment by age, comorbidities, nutritional status, proximity to index case and overcrowding, the risk of developing active TB among BCG vaccinated HHCs individuals with induction of mitochondrial damage was HR = 0.19 (95% CI: 0.1 to 0.5).

**Conclusions:**

The induction of monocytes mitochondrial damage by PPD stimulation correlates with protection of TB disease development in BCG-vaccinated HHCs. This represents a potential tool to predict susceptibility of developing active disease in this population.

## Introduction

The recent advances in the knowledge of *Mycobacterium tuberculosis*-host interactions and the immuno-pathogenesis of tuberculosis (TB) has led to further research on the possibility that some immunological parameters may predict the development of active disease among exposed individuals, such as contacts of smear positive TB patients [1, 2].

Several studies have focused on the search for new tools to detect active TB and also to identify infected individuals, the later representing a third of the world’s population [3]. Between 5 to 10% of infected people develop active TB, with half of them developing the disease during the first two years after exposure to a pulmonary TB case and the remaining at any time of their life span [1, 4]. Thus, it is reasonable to presume that most people develop a protective immune response which keeps the infection latent [5]. The chronicity of *Mycobacterium tuberculosis* (*M*.*tb*) infection and the natural course of the exposure to the bacilli, among many other variables, have made the diagnosis of latent-*M*.*tb* infection (LTBI) a complicated issue.

There is evidence that the mycobacteria may be eliminated by the host innate immune mechanisms [6] and the possibility of a spontaneous cure after the disease appears,it is unlikely to occur [7]. In addition, in immunocompromised people, the disease frequently develops after primary infection, as it occurs in HIV positive (+) patients, who have higher risk of developing active TB during the first year after exposure to the bacilli depending on the severity of immunodeficiency.

From an epidemiological standpoint, the study of factors that affect the progression from infection to the disease development is important, since their identification may result in the development of strategies that may lead to a more efficient control of the disease [8].

The precise mechanisms by which TB is reactivates in latently infected individuals are not known, although some of the factors that increase this risk have been considered [9]. Among them are the virulence of the bacillus, the intensity and time of exposure, and host factors such as age, sex and immune competence are well established [9]. This is why transmission of infection and progression to active disease is higher in households contacts (HHCs) living in the same dwelling with pulmonary TB cases [10].

One of the most relevant aspects of the study of host responses to *M*.*tb* infection is the antimicrobial functions of mononuclear phagocytes, including the mechanisms involved in death of the infected phagocytes [11]. Evidence accumulated during the last decade indicates that this type of cell death could serve as a potential biomarker for identifying the risk of TB disease progression among infected individuals [12, 13].

The pathways involved in triggering phagocytes’ death also play an important role in anti-*M*.*tb* effector mechanisms and TB pathogenesis, suggesting that a better understanding of cell death regulation may contribute to improving the immune effector responses and to develop new prophylactic and therapeutic interventions for TB control [13, 14]. There is evidence from animal models, human cells infected *in vitro* as well as biopsies from patients that mononuclear phagocytes´ death occurs during the course of infection. However, although the exact significance of the cells´ death in the development of the disease is still uncertain, the manipulation of cell death pathways will eventually affect the course of the infection [11, 15]. Although the interplay between the mononuclear phagocytes-apoptosis and the mycobacterial control is still under investigation, there is evidence that apoptosis pathways are able to trigger inflammatory mediators (pyroptosis) and autophagy which may serve to limit bacterial intracellular growth [16–19].

We have previously reported that monocyte cell death mechanisms could be related to the pathogenesis of TB. Specifically, we have observed that monocytes from TB patients are more prone to develop membrane damage, as occurs in necrosis, while monocytes isolated from healthy controls undergo a type of cell death that does not induce membrane damage [20, 21]. The aim of the present study is to determine whether the type of monocyte death (apoptosis or necrosis) might be used to predict the susceptibility of developing active TB in a cohort of HHCs. This is the first study that links the cell death mechanisms and susceptibility to the development of active TB at the population level.

## Materials and methods

### Type of study and population

Three hundred and sixty seven smear positive pulmonary TB patients and a cohort of 2,060 households contacts (HHCs) were recruited at the Medellín‘s metropolitan area (Colombia) from March 2005 to December 2006 and carefully followed during the next two to three years. The methodological aspects and the cohort study have already been extensively described [22].

### Ethics statement

The Ethics Committee of the Instituto de Investigaciones Médicas, Facultad de Medicina, of Universidad de Antioquia approved the project, as well as the health authorities from Medellín and the state of Antioquia as previously reported [22]. The detailed explanation about the ethics considerations that we took into account has been described [22]. Briefly, all the people who met the inclusion criteria were invited to participate, and those who agreed, signed the written consent form. Follow-up of participants was carried out by trained personnel that were unaware of the results of the cell death experiments. The local health authorities provided the treatment for those HHCs diagnosed with active tuberculosis, in agreement with Colombian health policies. Documents with personal information and blood samples were protected by codes specifically developed for this project [22].

### Monocyte death studies

**Fig 1:**
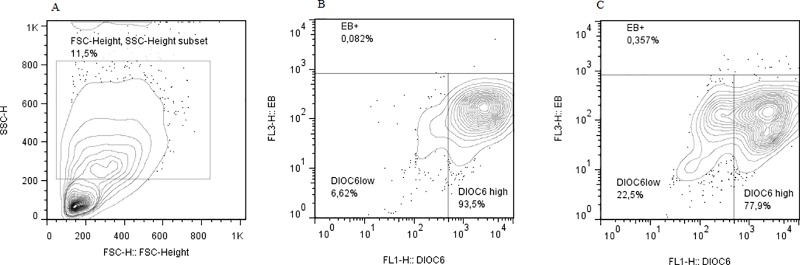
Flow cytometry strategy to determine mitochondrial and membrane damage on monocytes from PBMC. Panel A shows the FSC-H x SSC-H plot and the gate to select the monocyte population in cultured PBMCs. Panel B shows the DIOC6 x EB plot in monocytes from non-stimulated cultures. Panel C shows the DIOC6 x EB plot in monocytes PPD-stimulated cultures. Insets shows in panel A the percentage of cells within the monocyte gate, and in panels B and the percentage of cell with mitochondrial damage (DIOC6 low), membrane damage (EB+), or live cells DIOC6 high).

Peripheral blood mononuclear cells were obtained by centrifugation on Histopaque density gradients (Sigma-Aldrich, St Louis, MO, USA) for 30 min at 900 x g. The cells were washed with Phosphate Saline Dulbeco (DPBS, GIBCO, Life Technologies Grand Island, NY) and the viability (≥95%) was determined by trypan blue exclusion. Two hundred thousand peripheral blood mononuclear cells were resuspended in 1 mL of RPMI-1640 medium (Gibco, Grand Island, NY, USA) with 10% fetal bovine serum (FBS, BioWhittaker (Walkersville, MD, USA), stimulated or not with the Purified Protein Derivative of *Mycobacterium tuberculosis* (PPD. RT50, Copenhagen, Denmark) and cultured in 12 x 75 mm polystirene tubes for 48 h. After incubation, cells were stained with 700 nM DiOC_6_ to assess the changes in the state of mitochondrial permeability transition which is affected early during apoptosis and 5 μg/ml Ethidium Bromide (EB) to evaluate the cell membrane damage [15]. Cells were incubated at room temperature for 30 min, washed with DPBS to remove the excess of dye and 10,000 cells were analyzed in a flow cytometer EPICS XL (Beckman Coulter, Hialeah, FL). Based on Forward Scatter (FSC-H) and Side Scatter (SSC-H), cells corresponding to mononuclear phagocytes were gated to determine the percentages of cells with mitochondrial and/or cell membrane damaged **(Fig 1)**. Some samples were also stained with Annexin V and EB (data not shown), to determine whether PPD is able to induce phosphatidylserine exposure, which occurs mainly during apoptosis [23].

### Cell death index

In order to determine the effect of PPD on the mitochondrial or membrane damage the indexes of the cell damage levels were calculated as follows:

**Step 1:** The effect of PPD on mitochondrial damage was calculated as the proportion of cells with mitochondrial damage in response to PPD minus the proportion of cells with mitochondrial damage in absence of PPD stimulus (1.1).

*Level of mitochondrial damage = % of cells with mitochondrial damage in the PPD-stimulated cultures- % of cells with mitochondrial damage in the non-stimulated cultures* (1.1)

**Step 2:** From 1.1 it was assumed that any level above zero indicates an increase of mitochondrial damage in the stimulated cells, defining this as **PPD-induced mitochondrial damage**

**Step 3:** The effect of PPD on cell membrane damage, was determined by the proportion of cells with membrane damage in response to PPD minus the proportion of cells with membrane damage in absence of PPD stimulation (1.2).

*Level of membrane damage = % of cells with membrane damage in the PPD-stimulated cultures—% of cells with membrane damage in the non-stimulated cultures* (1.2).

**Step 4:** From 1.2 it was assumed that any level above zero indicates an increase of membrane damage in the stimulated cells, defining this as **PPD-induced membrane damage**.

### Other variables

The outcome in the HHCs was tuberculosis and its diagnosis was established as previously published [22]. The Colombian vaccination program uses the BCG-Japan strain. BCG vaccination was determined by the presence of the scar and confirmed in the case of children by their parents as previously reported [22]. The nutritional status was classified differently in children and adults. Normal nutritional status was defined as body mass index (BMI) ranging 18.5–25 kg/m^2^ in adults, and -1 Z score of weight and height ≤ 1 in children; underweight was defined as a BMI ≤ 18.5 kg/m^2^ in adults and in children with a Z score for age ≤ 1 and overweight as a BMI ≥ 25 kg / m^2^ in adults and children have a Z score > 1. In addition, the presence of any systemic, neurological, pulmonary, gastrointestinal, kidney, dermatological, or ophthalmological disease, previous transplantation or drug abuse was regarded as comorbidity. Rooms were tagged as overcrowded they housed more than 3 people per room [22].

### Statistical analysis

The effect of PPD on mitochondrial and cell membrane damage was evaluated using the Wilcoxon test. The frequencies and proportions were calculated for categorical variables and comparisons were done using the chi-square test or chi-square test for trend. To assess the factors associated with the increased apoptosis and necrosis, a logistic regression was performed using those variables in the bivariate analysis that showed a p-value < 0.20.

The odds ratio (OR) and the 95% confidence interval (CI) were adjusted by the generalized estimating equations (GEE), because the cohabitants shared the same exposure conditions that influenced the estimate of the associations. The model was estimated using a link logit function of a binomial probability distribution and an interchangeable correlation matrix. The incidence of tuberculosis was estimated for each of the variables, and the bivariate analysis included the estimated hazard ratio (HR) and 95% CI.

Since the stratified analysis identified an interaction between BCG scar and increased apoptosis, hence, those variables with a p-value < 0.20 in the log-rank test that met the proportional hazards assumption were included in a Cox regression, stratified by the presence of BCG scar. The survival curves were estimated using the Kaplan-Meier (KM) method.

To determine the predictive capacity of the cell death on disease progression, the maximum likelihood ROC Stata model was used, through the rocfit command. The software packages used were: SPSS release 22; Stata release 11.1; Statgraphics release 16.1.18 and Graph Pad Prism release 5.0.

## Results

Among the 2,060 HHCs, that were enrolled in this study, 57.2% were women and 34.8% were less than 15 years of age. Forty-Five percent lacked normal nutritional conditions: 18.6% were underweight and 26.4% were overweight. Nearly two thirds lived in low socioeconomic strata and 26.4% in overcrowded conditions.

### PPD stimulation induces mitochondrial and cell membrane damage

**Fig 2:**
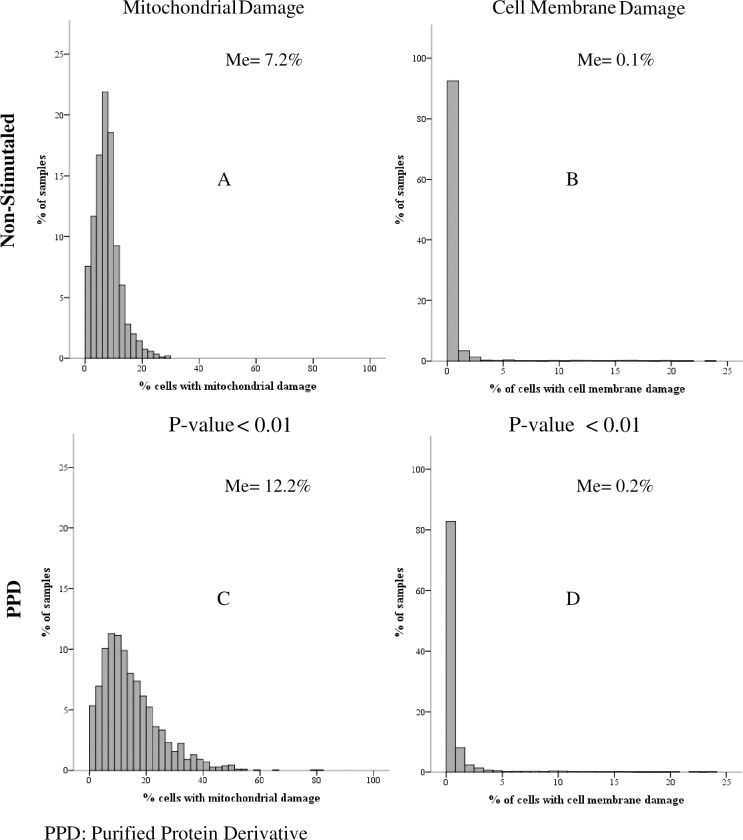
Distribution of the percentage of monocytes with mitochondrial damage and membrane damage in peripheral blood mononuclear cells (PBMC) cultures from household contacts stimulated and non-stimulated with PPD. The inset shows the Median (Me) of the percentage of monocytes with mitochondrial (Panels A and C) and cell membrane damage (Panels B and D).Stimulated and non-stimulated cultures were compared using the Wilcoxon test.

From a total of 2,060 blood samples obtained from the HHCs, 201 samples were excluded due to very low cell viability at the initiation of the experiment. From the remaining 1859 blood samples, mononuclear cells were stimulated or not with PPD. In non-stimulated cultures, the median percentage of mononuclear phagocytes with mitochondrial damage was 7.2% (IQR: 4.6–9.8%), while the median percentage of cells with cell membrane damage was 0.1% (IQR: 0–0.3%). In response to PPD, both the percentage of monocytes with mitochondrial damage (median 12.2%; IQR: 7.2 to 19.5%) and membrane damage (median 0.2%; IQR: 0 to 0.5%) increased significantly (p <0.01) **(Fig 2).**

**Fig 3:**
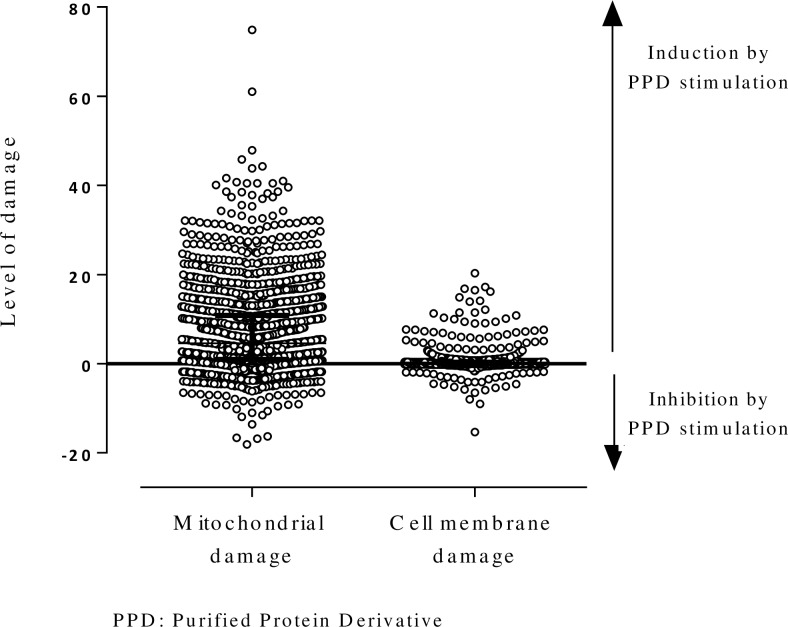
Effect of PPD stimulation on mitochondrial and cell membrane damage in mononuclear phagocytes from household contacts of smear-positive patients in Colombia. The level of mitochondrial damage was calculated as the difference between the percentage of PPD stimulated cells minus the percentage of non-stimulated cells for mitochondrial damage and cell membrane damage.

As defined in steps 2 and 4 (see Materials and Methods: **Cell Death Index**), in response to PPD, 83.4% monocytes (1151/1859) of the HHCs underwent mitochondrial damage and 50.9% (947/1859) underwent cell membrane damage. In some cases, the stimulation with PPD induced lower cell death compared to those of non-stimulated cells. In some individuals, there were monocytes with both membrane and mitochondrial damage **(Fig 3)**.

### Factors associated with damage to the mitochondria and cell membrane in response to PPD

The induction of mitochondrial damage had a positive association (p<0.05) with the following variables: low or middle socioeconomic level; exposure to index case for ≥ 168 hours and living in homes where ≥ 3 people slept in the same bedroom. The induction of cell membrane damage in response to PPD was higher in children under 4 years (p = 0.03), and HHCs who slept in the same household with the TB patients (p = 0.04) **(Table 1)**.

**Table 1:** Risk factors associated with the induction of monocytes’ mitochondrial and membrane damage in response to PPD, in mononuclear phagocytes obtained from household contacts of smear-positive TB patients in Colombia.

Factor	PPD-induced mitochondrial damage			PPD-induced membrane damage		
	OR^‡^	(IC95%)^¶^	P-value	OR^‡^	(IC95%)^¶^	P-value
**Gender**						
Female	1			1		
Male	1,20	1,0–1,5	0,13	0,97	0,8–1,2	0,78
**Age, years**						
≤ 4	1,15	0,7–1,8	0,55	1,57	1,1–2,4	0,03
5 14	0,93	0,7–1,3	0,65	1,20	0,9–1,5	0,12
15 or older	1			1		
**Socioeconomic status**						
I–II	6,68	1,9–23,0	0,003	0,51	0,3–0,8	0,01
III–IV	7,66	2,3–25,7	0,001	0,56	0,3–0,9	0,05
V–VI	1			1		
**BCG scar**						
No	1			1		
Yes	1,02	0,7–1,5	0,94	0,98	0,8–1,3	0,89
**Family history of TB**						
No	1			1		
Yes	0,88	0,6–1,3	0,49	0,97	0,7–1,3	0,82
**Comorbidities**†						
No	1			1		
Yes	0,92	0,6–1,4	0,67	0,87	0,7–1,1	0,29
**Nutritional status**						
Normal	1			1		
Underweight	0,78	0,5–1,2	0,21	0,98	0,8–1,3	0,91
Overweight	0,92	0,7–1,3	0,61	1,18	1,0–1,4	0,09
**Sputum bacterial load of index case**						
Weak (+)	1			1		
Moderate (++)	1,40	0,9–2,1	0,10	1,1	0,7–1,6	0,68
Strong (+++)	1,33	0,9–2,0	0,20	1,23	0,9–1,7	0,47
**Proximity to the index case**						
HHC slept in other household	1			1		
HHC slept in same household	0,86	0,5–1,4	0,53	1,54	1,0–2,3	0,04
HHC slept in same room	0,93	0,6–1,0	0,71	0,90	0,7–1,2	0,47
**Time the Index case remained in the house**						
≤ 84	1			1		
85–140	0,74	0,4–1,3	0,03*	1,27	0,9–1,9	0,62*
141–167	1,24	0,3–4,9		1,76	0,6–5,1	
168 y +	1,55	1,0–2,4		0,97	0,7–1,3	
**Persons per room**						
Less than 3	1			1		
3 or more	1,50	1,0–2,3	0,05	0,79	0,6–1,1	0,19

HHC: Household contacts. BCG: Bacillus Calmette-Guérin

^‡^ Odds ratio adjusted for other variables and GEE.

¶ 95% confidence interval

† Including consumption of toxic substances and drugs.

*Chi-square test for trend

There was no correlation between the production of IFN-γ induced by several mycobacterial antigens previously reported [22] and the PPD induced mitochondrial or membrane damage reported herein; however, in the sample of HHCs in whom TST was performed (491 HHCs), the induction of membrane damage was higher in the TST positive HHCs (53.5%) than in TST negative HHCs (40.6%) (p = 0.008).

### Mitochondrial damage predicts the susceptibility of the household contact of smear-positive TB patients to develop active disease

The overall incidence rate of tuberculosis during follow-up was 7.2 per 1,000 person-years (PY). The incidence was highest in children under 4 years of age, in HHCs patients who do not have BCG scar, in HHCs with some comorbidity (p ≤ 0.046), especially diabetic patients living in the same household (p ≤ 0.023) or in overcrowded households. The presence of BCG scar and excess weight had no significant protective effect against TB disease development. Tuberculosis incidence rates were similar in HHCs that showed induction of mitochondrial damage and cell membrane damage. However, the risk of developing the disease was higher in HHCs where the response to PPD showed no mitochondrial damage, compared with non-stimulated cultures (p ≤ 0.029) **(Table 2)**.

**Table 2:** TB incidence and non-adjusted Hazard Ratio (HR) that influences development of TB according to various factors related to households of smear-positive patients in Colombia.

Factor	n/N	Person-years (PY)	Incidence rate x (1000 PY)	Non-adjusted HR (95% CI)	P-value+
**PPD-induced mitocondrial damage**					
No	11/308	808,57	13,60	1	
Yes	23/1545	3897,31	5,90	0,45 (0,22–0,92)	0,029
**PPD-induced membrane damage**					
No	20/909	2275,05	8,79	1	
Yes	14/944	2430,82	5,76	0,67 (0,34–1,32)	0,243
**Age, years**					
≤ 4	7/181	451,5	15,51	2,05 (0,88–4,79)	0,127
5–14	4/467	1230,2	3,25	0,44 (0,15–1,27)	0,095
15 or more	23/1205	3024,3	7,61	1	
**BCG scar**					
No	12/388	987,9	12,15	1	
Yes	22/1428	3632,3	6,06	0,51 (0,25–1,03)	0,060
**Comorbidities**					
No	21/1469	3795,5	5,53	1	
Yes	8/265	655,0	12,21	2,30 (1,01–5,22)	0,046
**Diabetes Mellitus**					
No	30/1760	4486,9	6,69	1	
Yes	2/45	102,2	19,57	2,93 (0,70–1,23)	0,142
**Nutritional status**					
Normal	20/998	2538,5	7,88	1	
Overweight	5/489	1270,0	3,94	0,49 (0,18–1,31)	0,155
Underweight	9/340	841,9	10,69	1,33 (0,61–2,93)	0,474
**Proximity to index case**					
HHC slept in other household	20/1388	3516,7	5,69	1	
HHC slept in same household	7/189	479,7	14,59	2,73 (1,15–6,49)	0,023
HHC slept in same room	7/276	709,5	9,87	1,80 (0,76–4,27)	0,180
**Persons per room**					
Less than 3	21/1354	3454,8	6,08	1	
3 or more	13/486	1217,1	10,68	1,86 (0,93–3,74)	0,081

HR = Hazard Ratio. CI = Confidence Interval. BCG: Bacillus Calmette-Guérin. HHC: Household contacts

+ Log-rank test

**Fig 4:**
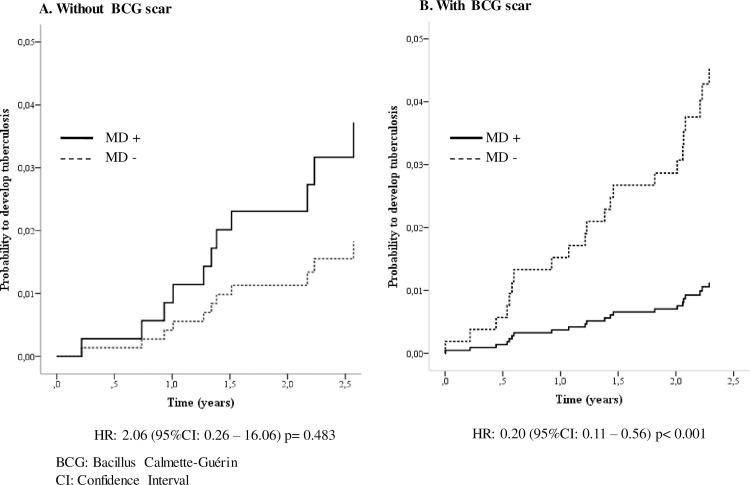
Effect of the induction of mitochondrial monocyte damage and BCG vaccination on the probability of developing tuberculosis in household contacts (HHC) of smear-positive TB patients. The dotted line shows the probability of the HHC that did not have mitochondrial damage (MD -); the black line shows the probability of HHC that did have mitochondrial damage (MD+). Household contacts of smear-positive patients were followed up for two years to detect development of active TB disease. Probabilities were calculated by Kaplan-Meier and compared by Log-Rank test. HR: Hazard Ratio.

In the HHCs without BCG scars in whom mitochondrial damage was induced in response to PPD, a trend to higher incidence of TB development was observed, however it did not attain statistical significance (HR: 2.06 95% CI 0.26–16.06) **(Fig 4A);** whereas, in HCCs with BCG scar and evidence of PPD induced mitochondrial damage significantly lower TB incidence was seen (HR: 0.20 95% CI: 0.11 to 0.56, p ≤ 0.001) **(Fig 4B)**.

**Fig 5:**
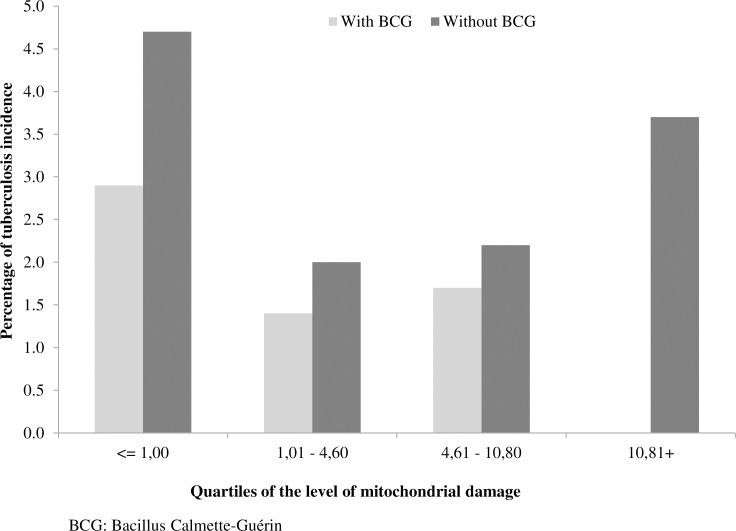
Incidence of tuberculosis according to the level of mitochondrial damage in household contacts of smear-positive patients with or without BCG vaccination. The level of mitochondrial damage was categorized by quartiles. The trend in decreasing incidence of active TB was assessed with chi square test for trend (chi square test for trend = 8.66, p = 0.003).

To further explore the effect of BCG vaccination on the PPD induction of mitochondrial damage and its association with TB incidence in the HHCs, the incidence was determined for the different quartiles of percentages of mitochondrial damage in the total population of HHCs, and in those with or without BCG scar. In the whole group of HHCs (chi square test for trend = 5.98, p = 0.015), and in those HHCs with BCG scar there was a significant decrease in TB incidence (chi square test for trend = 8.66, p = 0.003), at the point that in those BCG vaccinated HHCs located in the highest quartile of mitochondrial damage there were no incident cases; however, among the HHCs that had no BCG vaccination, there was no significant trend in TB incidence development associated with the level of mitochondrial damage **(Fig 5).**

The Cox regression analysis showed that induction of the mitochondrial damage in response to PPD was associated with significant level of protection against development of active TB in 81% of the HHCs positive for BCG scar (HR: 0.19 95% CI: 0.1–0.5) **(Table 3).**

**Table 3:** Risk factors associated with TB incidence according to the presence of BCG scar in household contacts of smear-positive TB patients in Colombia.

Factor	Without BCG scar		With BCG scar	
	(n = 354)		(n = 1313)	
	Adjusted HR^¶^	(95% CI)	Adjusted HR^¶^	(95% CI)
**PPD-induced mitochondrial damage**				
No	1		1	
Yes	1,65	(0,2–13,2)	0,19^*^	(0,1–0,5)
**Age, years**				
≤ 4	2,87	(0,6–14,3)	1,22	(0,3–4,5)
5–14	ND	ND	0,48	(0,2–1,6)
15 or more	1		1	
**Comorbidity**				
No	1		1	
Yes	1,17	(0,2–6,1)	3,06^*^	(1,1–8,2)
**Nutritional status**				
Normal	1		1	
Overweight	0,97	(0,2–4,5)	0,32	(0,1–1,5)
Underweight	1,46	(0,2–9,1)	1,17	(0,4–3,9)
**Proximity to the index case**				
HHC slept in other household	1		1	
HHC slept in same household	0,79	(0,1–7,2)	2,81^**^	(0,9–8,8)
HHC slept in same room	1,1	(0,2–5,8)	1,6	(0,5–5,3)
**Persons per room**				
Less than 3	1		1	
3 or more	2,61	(0,7–10,4)	2,03	(0,8–5,4)

ND = No data. HHC: Household contacts. BCG: Bacillus Calmette-Guérin

¶ Hazard Ratio adjusted by age, comorbidity, nutritional status, proximity of exposure to case and overcrowding

* p <0.05

** p <0.10

The presence of any comorbidity and a closer proximity to the index case during the early period with respiratory symptoms would significantly increase the risk of developing the disease among HHCs, while the induction of mitochondrial damage would significantly reduce the risk in HHCs exhibiting a BCG scar **(Table 3).** In HHCs without BCG scars had no statistically significant relationship with the development of TB.

**Fig 6:**
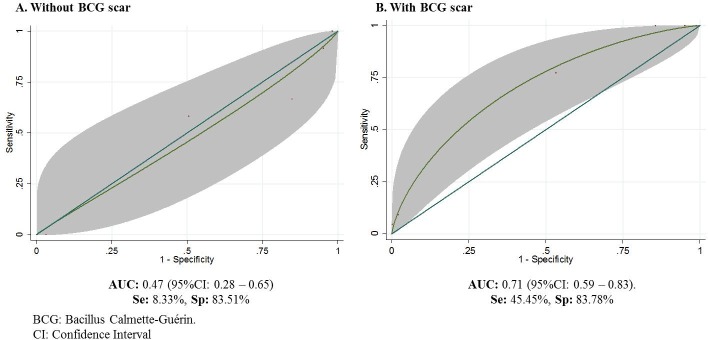
ROC curves showing the predictive capacity of the level of mitochondrial damage to predict active TB development in household contacts of smear-positive patients with or without BCG vaccination. The gray area shows the confidence interval of AUC (Area Under Curve),Se (Sensitivity), Sp (Specificity).

The ROC curves show the potential of using increased mitochondrial damage in monocytes to predict the disease development **(Fig 6).** The positive predictive value was higher when the BCG vaccination was present. Among the HHCs with BCG scar the AUC was 0.71 (95%CI: 0.59–0.83), while in those without BCG scar, the AUC was 0.47 (95%CI: 0.28–0.65).

## Discussion

Different mechanisms of cell death, particularly apoptosis and autophagy [24], have been presumed to play a protective role against a variety of pathogens [11]. When confronted with a pathogen that uses the host cellular resources for survival and replication, one strategy for defense is to activate host cell death programs. Host cell apoptosis is a well-established event in response to viral infections [25]. Many successful viral pathogens encode genes whose products suppress apoptosis of the host cell, thereby sustaining the niche for viral replication [26, 27]. Although, the extension of this paradigm to intracellular bacterial pathogens is more recent, a large number of cases have been identified with cell apoptosis, including mononuclear phagocytes’ death during mycobacterial infections [28]

Most of the published evidence points to apoptosis as an important macrophage response to *Mtb* infection [15, 19, 29–34], what remains unclear is its role in the pathogenesis of the disease [11] and whether the occurrence of any form of cell death could be used as a biomarker for predicting the susceptibility of developing an active TB.

This study provides evidence that increased mitochondrial damage in monocytes, an early event during apoptosis, may be associated with protection from the development of active tuberculosis in a population of individuals at high risk of infection. This is the first population-based study to provide evidence of an association between early events of cell mitochondrial and membrane damage and a protective effect from TB development [33, 35]. Several studies conducted using animal models and humans support the hypothesis that virulent strains of *M*. *tuberculosis* induce apoptosis [28, 32, 36, 37], while others argue that virulent mycobacteria strains inhibit it [38, 39]; however, none of these studies had sufficient statistical power to evaluate cell death and disease prognosis.

The findings presented here are based on information from a cohort of HHCs with close adherence to the research protocol and with a sufficiently large sample size to provide an adequate degree of precision and power to the estimates. Besides other immunological indicators that were explored in the cohort of the present study [22], an indicator of monocyte death was derived and validated to elucidate the role of this cellular response in the disease development. Analysis of monocyte death using this indicator is a novel approach that warrants comparison with available evidence on the topic.

In this study we found that the response of monocytes of HHCs of patients with pulmonary TB to PPD is characterized by a mixed profile of mitochondrial and membrane damage, similar to what was reported in patients with active TB [20]. These results are in agreement with Danelishvili *et al*., who proposed that the outputs of cell death are dependent on the cell background [28]. Our group had previously reported that peripheral blood monocytes from TB patients undergo apoptosis and necrosis *in vitro* when stimulated with PPD or infected with *M*. *tuberculosis* H37Rv, whereas monocytes from healthy controls do not. The mixed profile of apoptosis and necrosis[15], in the setting of high rates of infection and disease development [22], may be attributed in part to the high exposure to a TB case.

The factors associated with increased induction of mitochondrial and cell membrane damage reported herein, were similar to the factors that have been reported to be associated with an increased risk of latent infection [15]. Our findings revealed that HHCs that are younger, mostly impoverished and subjected to prolong and intense exposures to patients severe tuberculosis had more mitochondrial/membrane damage.

In this study, based on the dichotomy established in steps 2 and 4 of the methods section, it was found that in 83.4% of HHCs there was an induction of mitochondrial damage in response to PPD, while in the remaining 17%, PPD-stimulation appeared to be able to inhibit it. Some studies support this dual ability of *M*. *tuberculosis* to induce and to inhibit cellular responses. Increased apoptosis has been associated with host defense mechanisms [28] while its inhibition is a strategy that guarantees mycobacterial survival within the host cells [40]. Other studies propose that increased apoptosis is a mechanism employed by virulent strains to avoid the induction of macrophage cell death, hence becoming a key element for the survival of the bacteria inside the host [39] and to evade host immune response [41].

Prolonged exposure to the index case and overcrowded living spaces were among the factors that favored the induction of mitochondrial damage. The latter seems paradoxical taking into account that one of the most important conditions for early cellular responses to a noxious stimulus is its dependence on duration and severity of the injury [42]; mononuclear cells from HHC exposed for a short period of time may undergo apoptosis, but the highest bacterial load per cell may result in membrane damage causing death.

An important link between cell death and malnutrition was also observed. The absence of PPD-induced mitochondrial damage was common in underweight individuals (OR: 0.93 (95% CI: 0.7 to 1.3); while on the other hand, increasing membrane damage was associated with overweight (OR: 1.24 (95% CI: 1.0 to 1.5). It is well known that malnutrition negatively affects the cell-mediated responses which are considered the cornerstone of protective immunity against tuberculosis [43], and additionally obesity plays a significant role in membrane damage [44]. The information gathered also showed that most of the HHCs studied live in poverty and 26.7% of them (95% CI: 24.7 to 28.8) were overweight.

Our results show that the ability of PPD to induce monocyte mitochondrial damage correlates with protection of TB disease development in BCG vaccinated HHCs. The level of protection was 80% (adjusted HR: 0.20 95% CI: 0.1–0.5) among HHCs with BCG scars, while the risk level was 87% (HR: 1.87 95% CI: 0.2–15.2) among HHCs without BCG. The negative effect of the increased mitochondrial damage over active TB development among those without BCG scars could also be accounted for by the increase in monocyte viability due to BCG hindering apoptosis [45]. In addition, although an increase in mitochondrial damage clearly benefits the host in its fight against the mycobacteria still surviving and persisting within the tissues, it has been reported that in the later stages of infection, the mycobacteria use apoptosis to their advantage as they manage to successfully escape from apoptotic bodies and infect new cells which will die by necrosis [39].

The significant effect of apoptosis, not of necrosis, can apparently be explained by the finding that a large number of HHCs participating in this study showed this cell death phenotype [39]. Several studies have demonstrated that apoptosis of infected cells reduces the viability of the intracellular mycobacteria, whereas necrosis facilitates their spread to other cells and tissues [38, 40, 46]. However, no significant predictive capacity of necrosis induction on TB disease incidence was found within 2–3 years of follow-up period.

It would be possible to postulate that, since we used whole mononuclear cell cultures for PPD stimulation, the synergistic effect of BCG vaccination with induction of apoptosis maybe explained by a T-cell dependent recall response resulting in the production of cytokines which induces apoptosis and autophagy (ie, TNF-αα), where mitochondrial damage is an early event, and which are the mechanisms known to participate in mycobacterial control [17].

It is important to note that the lack of correlation between the induction of IFN-γγ previously reported in this HHC cohort [22] and the induction of mitochondrial or membrane damage reported in this study may be explained by the use of different stimuli: purified mycobacterial antigens in the former and PPD in the studies of cells death; this may be confirmed by the finding of an association of PPD-induced membrane damage with the TST response. However, further studies are needed to elucidate the mechanisms behind the interaction of BCG vaccination and the protective effect of apoptosis in individuals vaccinated with BCG. Studies exploring the prognostic value of cell death in different population groups, such as contacts of paucibacillary TB patients and the general population from endemic settings, will be useful to improve our understanding of its role on the outcome of those infected with *Mycobacterium tuberculosis*.

The result of the present study may be particularly relevant in countries with medium or high incidence of tuberculosis and that continues to use BCG vaccination, like Colombia that had an incidence rate of 36 cases per 100,000 population [47] and BCG vaccine coverage of 92.6% (98.2% in Medellín) as at 2008 when this study was conducted [48]. Elucidation of the immunological phenomena that influences the epidemiological outcomes of tuberculosis may contribute to further strengthen the TB control programs.
